# An Introduction to the Non-Equilibrium Steady States of Maximum Entropy Spike Trains

**DOI:** 10.3390/e21090884

**Published:** 2019-09-11

**Authors:** Rodrigo Cofré, Leonardo Videla, Fernando Rosas

**Affiliations:** 1 Centro de Investigación y Modelamiento de Fenómenos Aleatorios CIMFAV-Ingemat, Facultad de Ingeniería, Universidad de Valparaíso, Valparaíso 2340000, Chile; 2 Centre for Psychedelic Research, Department of Medicine, Imperial College London, London SW7 2DD, UK; 3 Centre for Complexity Science and Department of Mathematics, Imperial College London, London SW7 2AZ, UK; 4 Data Science Institute, Imperial College London, London SW7 2AZ, UK

**Keywords:** non-equilibrium steady states, maximum entropy principle, spike train statistics, entropy production

## Abstract

Although most biological processes are characterized by a strong temporal asymmetry, several popular mathematical models neglect this issue. Maximum entropy methods provide a principled way of addressing time irreversibility, which leverages powerful results and ideas from the literature of non-equilibrium statistical mechanics. This tutorial provides a comprehensive overview of these issues, with a focus in the case of spike train statistics. We provide a detailed account of the mathematical foundations and work out examples to illustrate the key concepts and results from non-equilibrium statistical mechanics.

## 1. Introduction

Being the brain one of the most complex systems within the observable universe, it is not surprising that there is still a large number of unanswered questions related to its structure and functions. With the aim of developing new ways of addressing such questions, there is an increasing consensus among neuroscientists in that interdisciplinary approaches are promising. As a prominent example of this, computational neuroscience has been greatly enriched during the last decades by tools, ideas and methods coming from statistical physics [1,2]. Moreover, these methods are recently being revisited with renewed interest due to the arrival of experimental techniques that generate huge volumes of data. In particular, neuroscientists have become progressively aware of the powerful computational techniques used by statistical physicists to analyze experimental data and large scale simulations.

When studying the firing patterns of collections of neurons, one of the most popular principles from statistical mechanics is the *maximum entropy principle* (MEP), which builds the least structured model that is consistent with average values measured from experimental data. These average values are usually restricted to firing rates and synchronous pairwise correlations, which gives rise to models composed by time independent and identically distributed (i.i.d) random variables, i.e., stochastic processes without temporal structure [3,4,5]. Needless to say, there exists strong evidence in favour of memory effects playing a major role in spike train statistics, and biological process in general [6,7,8,9]. Following this evidence, over the last years the study of complex biological systems has started to consider time-dependent processes where the past has an influence on future behavior [10,11,12]. The corresponding asymmetry between past and future is called the “arrow of time”, which is the unique direction associated with the irreversible flow of time that is noticeable in most biological systems.

Interestingly, the statistical physics literature has a fertile toolkit for studying time asymmetric processes [13]. First, one introduces the distinction between steady states that imply thermal equilibrium, and steady states that still carry fluxes—being called non-equilibrium steady states (NESS). Additionally, the extent to which a steady-state is not in equilibrium (i.e., the strength of its associated currents) can be quantified by the *entropy-production rate* [14], which is associated with the degree of time-irreversibility in the corresponding process [14]. Several studies have pointed out that being out-of-equilibrium is an important characteristic of biological systems [15,16,17]. Therefore, statistical characterizations consistent with the out-of-equilibrium condition should reproduce some degree of time irreversibility. One popular method that is suitable for studying these issues is Markov chain modeling [11,18,19,20,21,22].

Despite the potential of interdisciplinary pollination related to these fascinating issues, many scientists find it hard to explore these topics because of the major entry barriers, including differences in jargon, conventions, and notations across the various fields. To bridge this gap, this tutorial intends to provide an accessible introduction to the non-equilibrium properties of maximum entropy Markov chains, with an emphasis in spike train statistics. While not introducing novel material, the main added value of this tutorial is to present results of the field of non-equilibrium statistical mechanics in a pedagogical manner based on examples. These results have direct application to maximum entropy Markov chains, and may shed new light on the study of spike train statistics. This tutorial is suitable for researchers in the fields of physics or mathematics who are curious about the interesting questions and possibilities that computational neurosciences offers. The focus on this community is motivated by the growing community of mathematical physicists interested in computational neuroscience.

The rest of this tutorial is structured as follows. First, Section 2 introduces basic concepts of neural spike trains and Markov processes. Then, Section 3 introduces the notion of observable, and explores their fundamental properties. Section 4 introduces the core ideas of MEP, proposing the formal question and exploring methods for solving it. Section 5 studies various properties of interest of MEP models, including fluctuation-dissipation relationships, and their entropy production. Finally, Section 6 summarizes our conclusions.

## 2. Preliminary Considerations

This section introduces definitions, notations, and conventions that are used throughout the tutorial in order to give the necessary toolkit of ideas and notions to the unfamiliar reader.

### 2.1. Binning and Spike Trains

Consider a network of *N* spiking neurons, where time has been binned (i.e., discretized) in such a way that each neuron can exhibit no more than one action potential within one time bin $\Delta t_{b}$. Action potentials, or “spikes”, are “all-or-none” events, and hence, spike data can be encoded using sequences of zeros and ones. A spiking state is denoted by $x_{t}^{k}=1$, and corresponds to the event in which the *k*-th neuron spikes during the *t*-th time bin, while $x_{t}^{k}=0$ implies that it remains silent.

**Figure 1:**
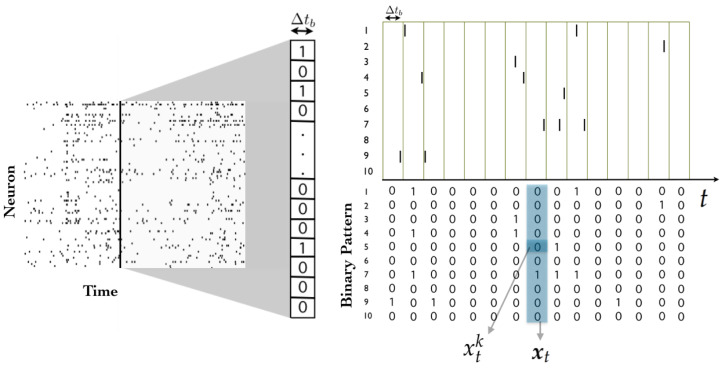
Illustration of a spike train, a spiking state and spike pattern. The time bin size $\Delta t_{b}$ determine the binary patterns.

A *spike pattern* is defined as the spike-state of all neurons at time bin *t*, and is denoted by $x_{t}:={\left[x_{t}^{k}\right]}_{k=1}^{N}$. A *spike block* is a consecutive sequence of spike patterns, denoted by $x_{t,r}:={\left[x_{s}\right]}_{s=t}^{r}$ (see Figure 1). While the length of the spike block $x_{t,r}$ is r-t+1, it is useful to consider spike blocks of infinite length starting from time t=0, which are denoted by x. Finally, in this tutorial we consider that a *spike train* is an infinite sequence of spiking patterns. This assumption turns out to be useful because it allows us to put our analysis in the framework of stochastic processes, and because it also allows us to characterize asymptotic statistical properties.

The set of all possible spike patters (or state space) in a network of *N* neurons is denoted by S, and the set of all spike blocks of length *R* in a network of *N* neurons is denoted by $S^{R}$.

Even at a single neuron level, for repetitions of the same stimulus, neurons respond randomly, but with a certain statistical structure. This is the main reason to look for statistical characterizations of spike trains. When trying to find a statistical representation considering a whole population of neurons responding simultaneously to a given stimulus, the problem is the following. Consider an experimental spike train from a network of *N* neurons where sequences of spike patterns are considered time-independent. The spike patterns can take $2^{N}$ values (state space). For N>10 is not possible to observe all possible states in real experimental data nor computer simulations (2 h of recordings binned at 20 ms produce less than $2^{19}$ spike patterns). For N=100 the state space is $2^{100}$, therefore the frequentist approach is useless to estimate the invariant measure. Can we learn something about the statistics of spike patterns from data having access only to a very small fraction of the state space? The maximum entropy principle provides an answer to this question. This principle has been used in the context of spike train statistics mainly considering firing rates and synchronous pairwise correlations, which gives rise to trivial stochastic processes composed by (i.i.d) random variables [3,4,5]. However, as mentioned in the introduction, there exists strong evidence in favour of past events playing a role in spike train statistics, and the biological process in general [6,7,8,9,11]. This principle can be generalized considering non-synchronous correlations, affording to build Markov chains from data. This approach opens the way to a richer modeling framework that can afford to model time irreversibility (highly expected in biological systems) and to a remarkable mathematical machinery based on non-equilibrium statistical mechanics which can be used to characterize collective behavior and to explore the capabilities of the system. We focus our tutorial on non-equilibrium steady states in the context of maximum entropy spike trains. In the next section of this tutorial we present the elementary properties of Markov chains (our main object of analysis) which will be used in the next chapters to extract relevant information about the underlying neuronal network generating the data.

### 2.2. Elementary Properties of Markov Chains

A stochastic process is a collection of random variables $X_{t}\in S$ indexed by t∈T that often refers to time. The set S represents the phase-space of the process; in the case of stochastic processes representing spike trains, one usually takes $S={\left\{0,1\right\}}^{N}$. Moreover, considering the temporal binning discussed in Section 2.1, usually T=N (the set of natural numbers) corresponds to the so-called discrete-parameter stochastic processes.

While spike trains can be characterized by stochastic processes dependent on an infinite past [23,24], Markov chains are particularly well-suited for modeling data sequences with finite temporal dependencies. In the next paragraphs we give the precise definition of a Markov process.

A stochastic process $\left(X_{t}:t\in N\right)$ defined on a measure space Ω is said to be a P–Markov chain if it satisfies the *Markov property* (with respect to the probability measure P): if, for every t∈N and for each sequence of states $x_{0},x_{1},\ldots ,x_{t+1}\in S$, the following relationship holds:

(1) $$\begin{array}{c} P\left(X_{t+1}=x_{t+1}|X_{0}=x_{0},X_{1}=x_{1},\ldots ,X_{t-1}=x_{t-1},X_{t}=x_{t}\right)=P\left(X_{t+1}=x_{t+1}|X_{t}=x_{t}\right)\;. \end{array}$$

This property is usually paraphrased as: the conditional distribution of the future given the current state and all past events depends exclusively on the current state of the process. It is direct to show that the Markov property is equivalent to the following condition: for every increasing sequence of indices $\left(i_{1}<i_{2}<\ldots <i_{n}\right)$ in N, and for arbitrary states $x_{i_{1}},x_{i_{2}},\ldots ,x_{i_{n}}$ in S, we have:

$$\begin{array}{c} P\left(X_{i_{n}}=x_{i_{n}}|X_{i_{n-1}}=x_{i_{n-1}},\ldots ,X_{i_{1}}=x_{i_{1}}\right)=P\left(X_{i_{n}}=x_{i_{n}}|X_{i_{n-1}}=x_{i_{n-1}}\right)\;. \end{array}$$

To characterize the transition probabilities, define a *S-indexed stochastic matrix* to be a doubly indexed array of non-negative real numbers P=(p(i,j):i,j∈S) such that $\sum_{j\in S}p\left(i,j\right)=1$ for every i∈S. It can be shown that a Markov chain is well-defined if the following is provided:

(i) An initial probability distribution, encoded by a vector $\mu :=(\mu_{i}:i\in S)$.
(ii) A collection of S-indexed stochastic matrices $\left\{P_{t}:={\left(p_{t}\left(i,j\right)\right)}_{i,j\in S}:t\in N\right\}$.

Using these two elements, one can build probability measures $P^{n}$ on $S^{n}$ as follows,

$$\begin{array}{c} P^{n}\left(i_{0},i_{1},\ldots ,i_{n-1}\right)=\mu \left(i_{0}\right)\prod_{j=0}^{n-2}P_{j}\left(i_{j},i_{j+1}\right)\;. \end{array}$$

Furthermore, the Kolmogorov extension theorem [25] guarantees the existence of a unique probability measure $P_{\mu }$ on $S^{N}$ such that the coordinate process satisfies:

$$\begin{array}{c} P_{\mu }\left(X_{0}=i_{0},X_{1}=i_{1},\ldots ,X_{n}=i_{n}\right)=P^{n}\left(i_{0},i_{1},\ldots ,i_{n}\right)\;, \end{array}$$

and with respect to which $\left(X_{t}:t\in T\right)$ is a Markov chain. In this case $P_{\mu }$ is said to be the probability law of the Markov chain $\left(X_{t}:t\in N\right)$. This notation also remarks that $P_{\mu }$ is the law with initial distribution μ.

### 2.3. Homogeneity, Ergodicity and Stationarity

A Markov chain is said to be *homogeneous* if the transition matrices do not depend on the time parameter *t*, i.e., if there exists a S-indexed stochastic matrix *P* such that $P_{t}=P$ for every t∈T. Note that if $\left(X_{t}:t\in T\right)$ is a P–homogeneous Markov chain, then for every t∈T:

(2) $$\begin{array}{c} P\left(X_{t+1}=j|X_{t}=i\right)=p\left(i,j\right):=p_{ij}\;. \end{array}$$

In the rest of this paper we focus exclusively on homogeneous Markov chains, since this is the model assumed in the maximum entropy framework.

Consider now a homogeneous Markov chain $\left(X_{t}:t\in T\right)$ with initial distribution μ and transition matrix *P*. Moreover, consider $p_{ij}^{\left(m\right)}$ to be the (i,j)-th entry of the product matrix $P^{m}=P\cdot P\cdot \ldots \cdot P$. These quantities correspond to the *m*–steps transition probabilities. Equation (2) can be generalized to

$$\begin{array}{c} P\left(X_{t+m}=j|X_{t}=i\right)=p_{ij}^{\left(m\right)}\;. \end{array}$$

A stochastic matrix *P* is said to be *ergodic* if there exists k∈N such that all the *k*–step transition probabilities are positive—i.e., there is a non-zero probability to go between any two states in *k* steps. A homogeneous Markov chain is ergodic if it can be defined by an initial distribution μ and an ergodic matrix.

Finally, a probability distribution π on S is called a *stationary distribution* for the Markov chain specified by *P* if

(3) $$\begin{array}{c} \pi P=\pi \;. \end{array}$$

Equivalently, π is stationary for *P* if π is a left eigenvector of the transition matrix corresponding to the eigenvalue λ=1, and is a probability distribution on S. While it is true that 1 is always an eigenvalue of *P*, it may be the case that no eigenvector associated to it can be normalized to a probability distribution. Further conditions for existence and uniqueness will be given in the next paragraph. Finally, if a S–indexed stochastic matrix *P* admits a stationary probability distribution π and $\left(X_{t}:t\in N\right)$ is a Markov chain with initial distribution π and transition matrix *P*, then for every t∈N and i∈S:

$$\begin{array}{c} P_{\pi }\left(X_{t}=i\right)=\pi_{i}\;. \end{array}$$

In this case $\left(X_{t}:t\in N\right)$ is said to be a stationary Markov chain, or that the Markov chain is started from stationarity.

The notion of homogeneous ergodic Markov chains is relevant in the context of spike train statistics because of the *Ergodic Theorem for finite-state Markov Chains*, which state that for all finite-state, homogeneous, ergodic Markov chains $\left(X_{t}:t\geq 0\right)$ with transition matrix *P* the following hold:

(a) There exists a unique stationary distribution π for *P* that satisfies that $\pi_{i}>0$ for every i∈S.
(b) For every j∈S,
  $$\begin{array}{c} \lim_{m\to +\infty }p_{ij}^{\left(m\right)}=\pi_{j}\;. \end{array}$$
  Equivalently, for every distribution ν, $\lim_{t\to \infty }P_{\nu }\left(X_{t}=j\right)=\pi_{j}\;.$ This property guarantees the uniqueness of the maximum entropy Markov chain.

### 2.4. The Reversed Markov Chain

Given a discrete ergodic Markov chain, it is mathematically possible to define its associated time reversed Markov chain. Some Markov chains in the steady-state yield the same Markov chain (in distribution) if the time course is inverted and others do not. It has been argued multiple times that those Markov chains that are different from their time inverted version are better suited to represent biological stochastic processes [6,7,9,11,12].

Let $\vec{P}$ be a stochastic matrix, and assume that it admits a stationary probability measure π. Assume too that $\pi_{i}>0$ for every i∈S (according to (a) in the Ergodic Theorem from the previous section, this is the case when $\vec{P}$ is ergodic). Define the S–indexed matrix $\overset{\leftarrow }{P}$ with entries:

$$\begin{array}{c} {\overset{\leftarrow }{P}}_{ij}=\frac{\pi_{j}}{\pi_{i}}{\vec{P}}_{ji}\;. \end{array}$$

A direct calculation shows that $\overset{\leftarrow }{P}$ is also a stochastic matrix. Moreover, if π is stationary for $\vec{P}$, then it is for $\overset{\leftarrow }{P}$ as well.

Using the above facts, let $P_{\pi }^{\to }$ and $P_{\pi }^{\leftarrow }$ be the laws of two stationary Markov chains, denoted by $X_{t}$ and $Y_{t}$, whose stationary distribution is π and transition probabilities are $\vec{P}$ and $\overset{\leftarrow }{P}$, respectively. The following holds

$$\begin{array}{cc} P_{\pi }^{\leftarrow }\left(Y_{0}=i_{0},Y_{1}=i_{1},\ldots ,Y_{n}=i_{n}\right) & =\pi_{i_{0}}{\overset{\leftarrow }{P}}_{i_{0}i_{1}}{\overset{\leftarrow }{P}}_{i_{1}i_{2}}\ldots {\overset{\leftarrow }{P}}_{i_{n-1}i_{n}}\\ \; & =\pi_{i_{0}}\frac{\pi_{i_{1}}}{\pi_{i_{0}}}{\vec{P}}_{i_{1}i_{0}}\frac{\pi_{i_{2}}}{\pi_{i_{1}}}{\vec{P}}_{i_{2}i_{2}}\ldots \frac{\pi_{i_{n}}}{\pi_{i_{n-1}}}{\vec{P}}_{i_{n}i_{n-1}}\\ \; & =\pi_{i_{n}}{\vec{P}}_{i_{n}i_{n-1}}{\vec{P}}_{i_{n-1}i_{n-2}}\ldots {\vec{P}}_{i_{1}i_{0}}\\ \; & =P_{\pi }^{\to }\left(X_{0}=i_{n},X_{1}=i_{n-1}\ldots ,X_{n}=i_{0}\right)\;. \end{array}$$

By virtue of this result, it is natural to call the chain $\left(Y_{t}:t\geq 0\right)$ the *reversed chain* associated to $\left(X_{t}:t\geq 0\right)$.

### 2.5. Reversibility and Detailed Balance

A transition matrix *P* is *reversible* with respect to π if the associated Markov chain started from π has the same law as the reversed chain started from the same distribution. The reversibility of *P* with respect to π is equivalent to the condition of *detailed balance*, given by

(4) $$\begin{array}{c} \pi_{i}P_{ij}=\pi_{j}P_{ji}\;\forall i,j\in S\;. \end{array}$$

Note that any probability measure π that satisfies detailed balance with respect to *P* is necessarily stationary, since

$$\begin{array}{c} \sum_{i\in S}\pi_{i}P_{ij}=\sum_{i\in S}\pi_{j}P_{ji}=\pi_{j}\sum_{i\in S}P_{ji}=\pi_{j}\;\mathrm{for}\;\mathrm{every}\;j\in S\;. \end{array}$$

The converse is, however, not true in general: a stationary distribution may not satisfy Equation (4).

Intuitively, Equation (4) states that, in the stationary state, the fluxes between each pair of states balance each other. In contrast, detailed balance is broken when there is a cycle of three or more states in the state space supporting a net probability current—even in the steady state. Detailed balance is also interpreted as “time reversibility”, as one could not distinguish the steady state dynamics of the system when going forward or backward in time. Certainly, this property is not expected in stochastic processes generated by biological systems. Several disciplines use the term “equilibrium” to refer to long-term behaviour, i.e., what is not transient. In this tutorial we use the term *equilibrium state* exclusively to refer to probability vectors that satisfy the detailed balance conditions—given in Equation (4). Markov chains that satisfy the detailed balance condition are referred as equilibrium steady states, and conversely, steady states that do not satisfy the detailed balance conditions are called *Non-Equilibrium Steady States* (NESS).

How to characterize (finite state, homogeneous) reversible Markov chains? Following [26], consider any finite graph $\left(S,{\left(c_{ij}\right)}_{i,j\in S}\right)$, with vertex set S and with the edge between vertices *i* and *j* labelled by the non-negative edge $c_{ij}=c_{ji}$. The graph can be visualized as a system of points labelled by S, and with a line segment between points whenever the corresponding conductance is positive. Define $c_{i}=\sum_{j\in S}c_{ij}$ and the S–indexed stochastic matrix given by

$$\begin{array}{c} p_{ij}=\frac{c_{ij}}{c_{i}}\;, \end{array}$$

Now define $C=\sum_{i\in S}c_{i}$. It is straightforward to prove that *P* is reversible with respect to the probability measure given by

$$\begin{array}{c} \pi_{i}=\frac{c_{i}}{C}\;, \end{array}$$

and thus it is stationary for *P*. The unique Markov chain started from π and transition matrix *P* is called the stationary random walk on the network $\left(S,{\left(c_{ij}\right)}_{i,j\in S}\right)$. Conversely, any reversible S–valued Markov chain can be identified with the random walk on the graph with vertex set S and edges given by $c_{ij}=c_{ji}=\pi_{i}p_{ij}$.

### 2.6. Law of Large Numbers for Ergodic Markov Chains

The Law of Large Numbers (LLN) that applies to independent and identically distributed random variables (i.i.d.) can be extended to the realm of ergodic Markov chains. In effect, for a given ergodic Markov chain $\left(X_{t}:t\geq 0\right)$ with stationary distribution π and transition matrix *P*, define the random variables $N_{i}^{\left(T\right)}$ equal to the number of occurrences of the state *i* up to time T-1, i.e.,

$$\begin{array}{c} N_{i}^{\left(T\right)}=\sum_{t=0}^{T-1}1_{\left\{X_{t}=i\right\}}, \end{array}$$

where $1_{\left\{\cdot \right\}}$ is an indicator function. Similarly, define the random variables $N_{ij}^{\left(T\right)}$ as the number of occurrences of the consecutive pair of states $\left(i,j\right)\in S^{2}$—in that order—up to time T-1, i.e.,

$$\begin{array}{c} N_{ij}^{\left(T\right)}=\sum_{t=1}^{T-1}1_{\left\{X_{t-1}=i,X_{t}=j\right\}}\;. \end{array}$$

With this, the *Strong Law of Large Numbers for Markov chains* can be stated as follows: if $\left(X_{t}:t\geq 0\right)$ is ergodic and π is its unique stationary distribution, then

$$\begin{array}{c} P_{\mu }\left(\lim_{T\to +\infty }\frac{N_{i}^{\left(T\right)}}{T}=\pi_{i}\right)=1\;\mathrm{and}\;P_{\mu }\left(\lim_{T\to +\infty }\frac{N_{ij}^{\left(T\right)}}{T}=\pi_{i}p_{ij}\right)=1, \end{array}$$

holds for any initial distribution μ. The result, in turn, implies the *Weak Law of Large Numbers for Markov chains*, which state that, following the above notation, for every ε>0 and for every starting distribution μ:

$$\begin{array}{c} \lim_{T\to +\infty }P_{\mu }\left(\left|\frac{N_{i}^{\left(T\right)}}{T}-\pi_{i}\right|>\varepsilon \right)=0\;\mathrm{and}\;\lim_{T\to +\infty }P_{\mu }\left(\left|\frac{N_{ij}^{\left(T\right)}}{T}-\pi_{i}p_{ij}\right|>\varepsilon \right)=0\;. \end{array}$$

Let’s denote by C(S) the space of real-valued functions on S. Clearly, any function of C(S) can be written as $f\left(x\right)=\sum_{i\in S}a_{i}1_{i}\left(x\right)$ for certain constants $a_{i}$, i∈S. Then, the above result generalizes as: for every f∈C(S), ergodic chain $X_{t}$, and probability distribution μ, the following holds:

$$P_{\mu }\left(\lim_{T\to +\infty }\frac{1}{T}\sum_{t=0}^{T-1}f\left(X_{t}\right)=E_{\pi }\left(f\left(X_{0}\right)\right)\right)=1\;.$$

This corresponds to a particular form of the Birkhoff Ergodic Theorem, which is briefly outlined in the next section and is relevant to characterize spike trains of observables as from data it is possible to accurately measure average values of firing rates and correlations. For an ergodic stationary Markov chain with a state space relatively small with respect to the sample size, this theorem guarantees that from a large sample the transition probabilities and the invariant measure can be recovered. This is not the case in spike train statistics at the population level as only a very small fraction of the state space is sampled in experimental spike trains. However, some features of the spike trains can be sampled very accurately from experimental data. In the next section, we present the basic elements to build from these characteristics a statistical model of the entire population.

## 3. Observables of Markov Chains and Their Properties

The notion of observable plays a central role in the study of maximum entropy spike trains. This section discusses their nature and fundamental properties.

### 3.1. Observables and Their Empirical Averages

Suppose a spiking neuronal network of *N* neurons is provided. Suppose too that measurements of spike patterns for *T* time bins have been performed. The observables are real-valued functions over the possible spike blocks, denoted here by $B:=S^{T}$. Let C(B) be the space of such observables, i.e., the linear space of real-valued functions f:B↦R. Recall the space C(S) of observables of range 1, discussed at the end of the above section. This space can be naturally embedded into C(B); thus, it can be considered as a linear subspace of the latter. More generally, the space of observables of range *R* for R≤T, denoted $C(S^{R})$, is just the space of real-valued functions on $S^{R}$, that we identify with its image through the natural embedding into C(B).

We are interested in the average of observables with respect to several probability measures. If μ is a probability measure on B (i.e., μ(ω)≥0 and $\sum_{\omega \in B}\mu \left(\omega \right)=1$) and *f* an observable of range R≤T i.e., $f\in C(S^{R})$, we define its expectation with respect to μ as

$$\begin{array}{c} \mu \left(f\right)=E_{\mu }\left\{f\right\}:=\sum_{\omega \in B}f\left(\omega \right)\mu \left(\omega \right). \end{array}$$

Since the space of blocks of length *T* is finite, the above sum is always finite, and thus our definition makes sense for every probability measure on B.

In the context of spike-trains, an important class of observable is made up of {0,1}-valued functions. It can be proved that any finite-range binary observable can be written as a finite sum of finite products of functions of the form $1_{\left\{X_{i}^{\left(j\right)}=1\right\}}$ that represents the event that the *j*-th neuron fires during the *i*-th bin. The average value if this observable is known as the firing rate of neuron *j*. This quantity has been proposed as one of the major neural coding strategies used by the brain [27].

Consider a spike block $x_{0,T-1}$, where *T* is the sample length. Although in most cases the probability measure μ that characterizes the spiking activity is not known, it is meaningful to use the experimental data to estimate the mean values of specific observables. The range of the validity of this procedure is usually based on prior assumptions about the nature of the source that originates the sample. For example, it can be assumed that the sample is a short piece of an infinite path that comes running from the far past, and so it can be assumed that this piece exhibits a behavior that is close to the stationary distribution. In this case, one can consider for any number R≤T the quantity:

$$\begin{array}{c} Q\left(y_{0},y_{1},\ldots ,y_{R-1}\right)=\sum_{j=0}^{T-R}1_{\left\{x_{j,j+R-1}=\left(y_{0},y_{1},\ldots ,y_{R-1}\right)\right\}}, \end{array}$$

that counts the number of appearances of the sequence $\left(y_{0},\ldots ,y_{R-1}\right)$ as a consecutive sub-sequence of $x_{0,T-1}$. Now, for any set $A\subseteq S^{R}$, define:

$$\begin{array}{c} \mu_{x_{0,T-1}}\left(A\right):=\frac{1}{T-R+1}\sum_{y\in A}Q\left(y\right), \end{array}$$

$$\mu_{x_{0,T-1}}\left(f\right)=A_{T}\left(f\right)=\frac{1}{T-R+1}\sum_{i=0}^{T-R}f\left(x_{i,R-1+i}\right).$$

When the empirical distribution is not explicitly stated, it is customary to write 〈f〉 to denote the average of the observable *f* with respect to this probability measure.

### 3.2. Moments and Cumulants

Observables are random variables whose average values can be determined from experimental data or from the explicit representation of the underlying measure characterizing the stochastic process generating the data. Important statistical properties of random variables are encoded in the *cumulants*. We will use the cumulants in Section 5 of this tutorial to characterize and infer properties of maximum entropy Markov chains. Let us now introduce them.

The moment of order *r* of a real-valued random variable *X* is given by $m_{r}=E\left(X^{r}\right)$, for r∈N (here we freely use the notation E to denote the expectation with respect to a probability measure that should be inferred from the context). The moment generating function (or Laplace transform) of a random variable is defined by:

$$\begin{array}{c} M\left(t\right)=E\left(e^{tX}\right), \end{array}$$

and provided it is a function of *t* with continuous derivatives of arbitrary order at 0, we have that:

$$\begin{array}{c} m_{r}={\left(\frac{d^{r}}{dt^{t}}M\right)}_{t=0}. \end{array}$$

The cumulants $\kappa_{r}$ are the coefficients in the Taylor expansion of the cumulant generating function. The cumulants are defined as the logarithm of the moment generating function, namely,

$$\begin{array}{c} \ln M\left(t\right)=\sum_{r}\kappa_{r}t^{r}/r!\;. \end{array}$$

The relation between the moments and cumulants is obtained extracting coefficients from the Taylor expansion, i.e.,

(5) $$\begin{array}{c} \kappa_{r}={\left(\frac{d^{r}}{dt^{r}}\ln\left(M\left(t\right)\right)\right)}_{t=0} \end{array}$$

which yields the first values:

$$\begin{array}{ccc} \kappa_{1} & = & m_{1}\;,\\ \kappa_{2} & = & m_{2}-m_{1}^{2}\;,\\ \kappa_{3} & = & m_{3}-3m_{2}m_{1}+2m_{1}^{3}\;,\\ \kappa_{4} & = & m_{4}-4m_{3}m_{1}-3m_{2}^{2}+12m_{2}m_{1}^{2}-6m_{1}^{4}, \end{array}$$

and so on. In particular, the first four cumulants are the mean, the variance, the skewness and the kurtosis.

### 3.3. Observables and Ergodicity

Let θ:Ω↦Ω be the shift operator that acts on a sequence ω∈Ω as:

$$\begin{array}{c} {\left(\theta \left(\omega \right)\right)}_{i}=\omega_{i+1}, \end{array}$$

i.e., θ shifts the sequence one position to the left. Now, assume that the Markov chain $\left(X_{t}:t\geq 0\right)$ is ergodic. Let π be its unique stationary probability distribution. The Birkhoff Ergodic Theorem states that under the above assumptions, for every f∈C(B):

$$\begin{array}{c} P_{\mu }\left(\lim_{N\to +\infty }\frac{1}{N}\sum_{n=0}^{N-1}f\circ \theta^{n}=E_{\pi }\left(f\right)\right)=1, \end{array}$$

for every initial measure μ. This equation means that under the ergodic hypothesis, the temporal averages converge to the spatial averages. The importance of this fundamental result should not be underestimated since this result supports the practice of regarding averages of (hopefully) large samples of experimental data as faithful approximations of the *true* values of the expectations of the observables.

### 3.4. Central Limit Theorem for Observables

Consider an arbitrarily large sequence of spike patterns of *N* neurons. Consider t∈N and let $x_{0,t-1}$ be the spike-block of length *t*. Also, let *f* be an arbitrary observable of fixed range *R*. The asymptotic properties of $A_{t}\left(f\right)$ are established in the following context: the finite sample is drawn from an ergodic Markov chain, i.e., $x∼P_{\nu }$, where $P_{\nu }$ is the Markov probability measure of an ergodic chain $\left(X_{t}:t\geq 0\right)$ started from an arbitrary initial distribution. Let π be the unique stationary measure for the Markov chain. Observe that by virtue of the ergodic assumption, the empirical averages of observables become more accurate as the sampling size grows, i.e.,

$$\begin{array}{c} P_{\nu }\left(A_{t}\left(f\right)\to E_{\pi }\left\{f\right\}\right)=1. \end{array}$$

for any starting condition ν. However, the above result does not clarify the rate at which the accuracy improves. The central limit theorem (CLT) for ergodic Markov chains provides a result to approach this issue (for datails see [28]).

**Theorem** **1** **(Central** **limit** **theorem** **for** **ergodic** **Markov** **chains).** *Under the above assumptions, and keeping notation, define:*

$$\begin{array}{c} \sigma =\sqrt{\left(E_{\pi }((f\left(X_{0},\ldots ,X_{R-1}\right)-E_{\pi }{\left(f\left(X_{0},\ldots ,X_{R-1}\right)\right)}^{2}\right)}\;. \end{array}$$

*Let $L_{t}$ be the law of the random variable $\frac{\sqrt{t}}{\sigma }\left[A_{t}\left(f\right)-E_{\pi }\left\{f\right\}\right]$ under the measure $P_{\nu }$ of an ergodic Markov chain started from an arbitrary distribution. Let L be the law of a standard normal random variable. Then $L_{t}\to L$ in the sense of weak convergence of convergence in distribution. This is usually written as:*

$$\begin{array}{c} P_{\nu }\left\{\frac{\sqrt{t}}{\sigma }\left[A_{t}\left(f\right)-E_{\pi }\left\{f\right\}\right]\leq x\right\}\to \frac{1}{\sqrt{2\pi }\sigma }\int_{-\infty }^{x}e^{-\frac{s^{2}}{2\sigma }}ds. \end{array}$$

This theorem implies that “typical” fluctuations of $A_{t}\left(f\right)$ around its long term average $E_{\pi }\left\{f\right\}$ are of the order of $\sigma /\sqrt{t}$. For spike trains, this theorem quantifies the expected Gaussian fluctuations of observables in terms of the sample size of the experimental data.

### 3.5. Large Deviations of Average Values of Observables

Although the CLT for ergodic Markov chains is precise in describing the typical fluctuations around the mean, it does not characterize the probabilities of large fluctuations. While it is clear that the probability of large fluctuations of average values vanish as the sample size increases, it is sometimes relevant to characterize the decrease rate of this probability. That is what the large deviation principle (LDP) does.

Let *f* be a function of finite range defined on the space of sequences. In many situations, *f* will be a {0,1}–valued function. Let $P_{\pi }$ be the probability measure on the space of sequences induced by an ergodic Markov chain with stationary probability π. The empirical average $A_{t}\left(f\right)$ satisfies a large deviation principle (LDP) with rate function $I_{f}$, defined as

(6) $$I_{f}\left(s\right):=-\lim_{t\to \infty }\frac{1}{t}\log P_{\pi }\left\{A_{t}\left(f\right)>s\right\},$$

if the above limit exists. The above condition implies for large *t* that $P_{\pi }\left\{A_{t}\left(f\right)>s\right\}\approx e^{-tI_{f}\left(s\right)}$. In particular, if $s>E_{\pi }\left\{f\right\}$ the Law of Large Numbers (LLN) ensure that $P_{\pi }\left\{A_{t}\left(f\right)>s\right\}$ goes to zero as *t* increases, but the rate function quantifies the speed at which this occurs.

Calculating $I_{f}$ using the definition (Equation (6)), is usually impractical. However, the Gärtner-Ellis theorem provides a clever alternative to circumvent this problem [29]. Let us introduce the *scaled cumulant generating function* (SCGF) associated to the random variable *f* by

(7) $$\lambda_{f}\left(k\right)=:\lim_{t\to \infty }\frac{1}{t}\ln E_{\pi }\left[e^{tkA_{t}\left(f\right)}\right],\;k\in R,$$

when the limit exists (details about cumulant generating functions are found in [30]). While the empirical average $A_{t}\left(f\right)$ is taken over a sample (empirical measure), the expectation in (7) is computed over the probability distribution given by $P_{\pi }\left\{\cdot \right\}$.

**Theorem** **2** **(Gärtner-Ellis** **theorem).** *If $\lambda_{f}$ is differentiable, then the average $A_{t}\left(f\right)$ satisfies a LDP with rate function given by the Legendre transform of $\lambda_{f}$, that is*

(8) $$I_{f}\left(s\right)=\max_{k\in R}\left\{ks-\lambda_{f}\left(k\right)\right\}.$$

Therefore, the large deviations of empirical averages $A_{t}\left(f\right)$ can be characterized by first computing their SCGF and then finding their Legendre transform.

A useful application of the LDP is to estimate the likelihood that the empirical average $A_{t}\left(f\right)$ takes a value far from its expected value. Let us assume that $I_{f}\left(s\right)$ is a positive differentiable convex function. Then, $\lambda_{f}\left(k\right)$ is also differentiable [31] (for a comprehensive discussion about the differentiability of $\lambda_{f}\left(k\right)$ see [30].) Then, as $I_{f}\left(s\right)$ is convex it has a unique global minimum. Denoting this minimum by $s^{*}$, from the differentiability of $I_{f}\left(s\right)$ it follows that $I_{f}\left(s^{*}\right)=0$. Additionally, it follows from properties of the Legendre transform that $s^{*}=\lambda_{f}^{\prime }\left(0\right)=E_{p}\left\{f\right\}$, which is the LLN that says that $A_{t}\left(f\right)$ concentrates around $s^{*}$. Consider $s\neq s^{*}$ and that $I_{f}\left(s\right)$ admits a Taylor expansion around $s^{*}$

$$I_{f}\left(s\right)=I_{f}\left(s^{*}\right)+I_{f}^{\prime }\left(s^{*}\right)\left(s-s^{*}\right)+\frac{I_{f}^{\prime \prime }\left(s^{*}\right){\left(s-s^{*}\right)}^{2}}{2}+O{\left(s-s^{*}\right)}^{3}\;.$$

As $s^{*}$ is zero and a minimum of I(s), the first two terms of this expansion are zero, and as I(s) is convex $I^{\prime \prime }\left(s\right)>0$. For large *t*, it follows from (6) that

(9) $$\begin{array}{cc} p\{A_{t}\left(f\right)>s\} & \approx e^{-tI_{f}\left(s\right)}\\ \; & \approx e^{-t\left(\frac{I_{f}^{\prime \prime }\left(s^{*}\right){\left(s-s^{*}\right)}^{2}}{2}\right)}\;, \end{array}$$

so the “small deviations” (we are using Taylor expansion) of $A_{t}\left(f\right)$ around $s^{*}$ are Gaussian (in Equation (9) $1/I_{f}^{\prime \prime }\left(s^{*}\right)=\lambda_{f}^{\prime \prime }\left(0\right)=\sigma^{2}$). In this sense, the LDP can be considered as an extension of the CLT as it goes beyond the small deviations around $s^{*}$ (Gaussian), but additionally the large deviations (not Gaussian) of $A_{t}\left(f\right)$.

## 4. Building Maximum Entropy Temporal Models

This section presents the main concepts behind the construction of maximum entropy models for temporal data. The next Section 4.1, introduces the concept of entropy, and then Section 4.2 formulates the problem of maximizing the entropy rate. Methods for solving this problem are discussed in Section 4.3, which are then illustrated in an example presented in Section 4.4.

### 4.1. The Entropy Rate of a Temporal Model

#### 4.1.1. Basic Definitions

In order to give mathematical meaning to the rather vague notion of uncertainty, a natural approach is to employ the well-established notion of *Shannon entropy*. For any probability measure *p* defined over the state space *E* (not necessarily S), the Shannon entropy of *p* is given by

$$S\left[p\right]:=-\sum_{x\in E}p\left(x\right)\log p\left(x\right)\;.$$

Note that this definition can be used for measures on the spaces of infinite sequences $E^{N}$. However, as in most cases of interest, the value saturates in infinite. A better suited notion in this context is given by the *entropy rate*, which plays a crucial role in the rest of this tutorial.

**Definition** **1** **(entropy** **rate).** *Let μ be a probability measure on the space of sequences $S^{N}$. For n≥1 let $\mu_{n}$ be the probability measure induced by μ on the initial n coordinates, i.e., $\mu_{n}$ is the probability distribution on $E^{n}$ given by:*

$$\begin{array}{c} \mu_{n}\left(x_{0},x_{1},\ldots ,x_{n}-1\right)=\mu \left(\omega \in S^{N}:X_{i}=x_{i}\;\mathrm{for}\;i=0,1,\ldots ,n-1\right). \end{array}$$

*The entropy rate of the measure μ is defined by:*

(10) $$S\left[\mu \right]=\lim_{n\to \infty }\frac{1}{n}S\left[\mu_{n}\right].$$

The above definition applies to any probability distribution on the space of sequences. Intuitively, the entropy rate correspond to the entropy per time unit, and represents how much “uncertainty” is created by the process as time moves forward.

#### 4.1.2. The Entropy Rate of I.I.D. and Markov Models

Let us consider first a null model of spike activity, where there is complete statistical independence between two consecutive spike patters. For this, first recall that $S={\left\{0,1\right\}}^{N}$, where *N* is the fixed number of neurons. Without loss of generality, we can enumerate the elements of S as $s_{1},s_{2},\ldots ,s_{2^{N}}$. Let $\nu =(\nu_{1},\nu_{2},\ldots ,\nu_{2^{N}})$ be a probability measure on S such that:

$$\begin{array}{c} \nu \left(s_{k}\right)=\nu_{k} \end{array}$$

For a *T*–block $x=\left(x_{0},x_{1},\cdots ,x_{T-1}\right)\in S^{T}$ and for every s∈S, we set:

$$\begin{array}{c} N_{s}^{T}\left(x\right)=\sum_{i=0}^{T-1}1_{\left\{x_{i}=s\right\}}. \end{array}$$

On the space of infinite spike trains $S^{N}$ we consider the probability $\mu =\nu^{⊗N}$, i.e., the product measure on the space of spike trains. Observe that the induced measure is given by:

$$\begin{array}{c} \mu_{n}\left(x_{0},x_{1},\ldots ,x_{T-1}\right)=\prod_{k=1}^{2^{N}}\nu_{k}^{N_{s}^{t}\left(x_{0},\ldots ,x_{T-1}\right)}\;. \end{array}$$

With this, a straightforward calculation shows that

$$\begin{array}{c} S\left[\mu \right]=S\left[\nu \right]=-\sum_{k=1}^{2^{N}}\nu_{k}\ln\left(\nu_{k}\right)\;, \end{array}$$

and in this case we observe that the entropy rate is equal to the entropy of the probability distribution induced by each coordinate map.

A reasonable next step in the hierarchy of models is to weaken the independence hypothesis and assume instead that the spike activity keeps some bounded memory of the past. For this, following the considerations of Section 2, let us consider an ergodic discrete Markov chain with transition matrix *P* and invariant distribution π taking values in S. Let μ=μ(P,π) be the measure induced by this chain on the space $S^{N}$. Observe that, with the above notation:

$$\begin{array}{c} \mu_{n}\left(x_{0},x_{1},\ldots ,x_{n-1}\right)=\pi_{x_{0}}\prod_{j=1}^{n-1}P_{x_{j-1}x_{j}}. \end{array}$$

A direct computation shows that

$$\begin{array}{cc} S[\mu_{1}] & =-\sum_{\left(x_{0},x_{1}\right)\in S^{2}}\pi_{x_{0}}P_{x_{0}x_{1}}\ln\left(\pi_{x_{0}}P_{x_{0}x_{1}}\right)\\ \; & =-\sum_{x\in S}\pi_{x}\ln\left(\pi_{x}\right)-\sum_{\left(x_{0},x_{1}\right)\in S^{2}}\pi_{x_{0}}P_{x_{0}x_{1}}\ln\left(P_{x_{0}x_{1}}\right), \end{array}$$

and induction shows that:

$$\begin{array}{c} S\left[\mu_{n}\right]=-\sum_{x\in S}\pi_{x}\ln\left(\pi_{x}\right)-n\sum_{\left(x_{0},x_{1}\right)\in S^{2}}\pi_{x_{0}}P_{x_{0}x_{1}}\ln\left(P_{x_{0}x_{1}}\right). \end{array}$$

Thus dividing by *n* and taking the limit in Equation (10), one finds that

$$\begin{array}{c} S\left[\mu \right]=-\sum_{\left(x_{0},x_{1}\right)\in S^{2}}\pi_{x_{0}}P_{x_{0}x_{1}}\ln\left(P_{x_{0}x_{1}}\right). \end{array}$$

### 4.2. Entropy Rate Maximization under Constraints

Now we introduce the central problem of this tutorial. Assume we have empirical data from spiking activity. Consider the empirical averages of *K* observables, $\langle f_{k}\rangle$, for $f_{k},k=1,\ldots ,K$. We need to characterize the Markov chains that are consistent with these average values. Except for trivial and uninteresting situations, there is no finite set of empirical averages that uniquely determines a distribution μ on $S^{N}$ that fits the averages, in the sense that

$$\begin{array}{c} \mu \left(f_{k}\right)=\langle f_{k}\rangle \;\mathrm{for}\;k=1,\ldots ,K. \end{array}$$

Consequently, we need to impose further restrictions in order to guarantee uniqueness. A useful and meaningful approach is the so-called Maximum Entropy Markov Chain model (MEMC), which fit the unique probability measure μ among all the stationary Markov measures ν on $S^{N}$ that match the expected values of a given set of observables and that maximizes the entropy rate. Mathematically, it is written in the following form:

$$\begin{array}{cccc} \; & \max_{\nu \in M_{inv}}\; & \; & S[\nu ]\\ \; & \mathrm{subject}\;\mathrm{to}\; & \; & \nu \left(f_{k}\right)={\langle f_{k}\rangle }_{e}=C_{k},\;\forall k\in \left\{1,\cdots ,K\right\}, \end{array}$$

where $M_{inv}$ is a shorthand for the sets of stationary Markov measures on $S^{N}$. Formally:

$$\begin{array}{c} M_{inv}:=\left\{\left(\pi ,P\right):\pi \;\mathrm{is}\;a\;\mathrm{probability}\;\mathrm{on}\;S,P\;\mathrm{is}\;\mathrm{stochastic},\;\pi P=\pi \right\}. \end{array}$$

It is to be noted that the maximum entropy principle can be derived in some scenarios from more general principles based on large deviation theory [30]. In this framework, entropy maximization corresponds to Kullback-Leiber divergence minimization. This approach can be useful for accounting additional information that is not in the form of functional constraints, but as a Bayesian prior. A major drawback of this approach to be applied to spike trains, is that it assumes stationarity in the data. While this condition is not to be naturally expected in biological systems, controlled experiments can be carried out in the context of spike train analysis in order to maintain these conditions [3,8,32,33]. The maximum entropy principle as presented here is useful only in the stationary case. However, some extensions have been proposed [34,35]. Note also that there are alternative variational principles which can be used to find distributions that extremize the value of quantities such as the maximum entropy production principle [36,37,38], or the Prigogine minimum entropy production principle [39,40]. To the best of our knowledge, these alternatives have not yet been explored in the context of spike train statistics.

### 4.3. Solving the Optimization Problem

We now discuss techniques for finding models that maximize the entropy rate.

#### 4.3.1. Lagrange Multipliers and the Variational Principle

To solve the above optimization problem, let us introduce the set of Lagrange multipliers $h_{k}\in R$ and an *energy* function $H=\sum_{k=1}^{K}h_{k}f_{k}$, which is a linear combination of observables. Consider the following unconstrained optimization problem, which can be framed in the context of the *variational principle* of the thermodynamic formalism [41]:

(11) $$F\left[H\right]=\underset{\nu \in M_{inv}}{\sup}\left\{S[\nu ]\;+\;\nu (H)\right\}=S\left[\mu \right]\;+\;\mu \left(H\right),$$

where F[H] is called the *free energy* and $\nu \left(H\right)=\sum_{k=1}^{K}h_{k}\;\nu \left(f_{k}\right)$ is the average value of H with respect to the measure ν. The following holds:

$$\frac{\partial F[H_{h}]}{\partial h_{k}}=E_{p}\left\{f_{k}\right\}=C_{k},\;\forall k\in \left\{1,...,K\right\},$$

where $E_{p}\left\{f\right\}$ is the average of $f_{k}$ with respect to *p* (maximum entropy measure), which is equal (by restriction) to the average value of $f_{k}$ with respect to the empirical measure from the data.

The maximum-entropy (ME) principle [42] has been successfully applied to spike data from the cortex and the retina [3,8,9,11,12,43]. The approach starts by fixing the set of constraints determined by the empirical average of observables measured from spiking data. Maximizing the entropy (concave functional) under constraints, gives a unique distribution. The choice of observables to measure in the empirical data (constraints) determines the statistical model. The approach of Lagrange multipliers may not be practical when trying to fit a MEMC. In the next section we introduce an alternative optimization based on spectral properties.

#### 4.3.2. Transfer Matrix Method

In order to illustrate the transfer matrix method, we start with a classical example that allow us to introduce a fundamental definition. Let *A* be a adjacency matrix i.e., a {0,1}-valued square matrix with rows and columns indexed by the elements of S. If there exists an n≥0 such that

$$\begin{array}{c} A_{ij}^{n}>0 \end{array}$$

for every i,j∈S, we say that *A* is *primitive*. The next well-known theorem of Linear Algebra is crucial [44] for the uniqueness of the MEMC.

**Theorem** **3** **(Perron-Frobenius** **theorem).** *Let A be a primitive matrix. Then,*

- *There is a positive maximal eigenvalue ρ > 0 such that all other eigenvalues satisfy $|\rho^{\prime }|<\rho$. Moreover ρ is simple;*
- *There are positive left- and right-eigenvectors $u=\left(u_{1},\cdots ,u_{k}\right),v=\left(v_{1},\cdots ,v_{k}\right)$ s.t. uA=ρu,Av=ρv.*

Apply the above theorem to a primitive matrix *A*, and define:

$$P_{ij}=\frac{A_{ij}v_{j}}{\rho v_{i}};\;\pi_{i}=\frac{u_{i}v_{i}}{\langle u,v\rangle },$$

where 〈u,v〉 is the standard inner product in $R^{2^{N}}$ (we refer the reader to [44] for details). The matrix *P* built above is stochastic. Moreover, π is its unique stationary measure. Define the Parry measure to be the Markov measure:

$$\begin{array}{c} \mu \left(i_{0},i_{1},\cdots ,i_{n}\right)=\pi_{i_{0}}P_{i_{0}i_{1}},\cdots ,P_{i_{n-1}i_{n}}. \end{array}$$

It is well known that the Parry measure is the unique measure of maximal entropy consistent with the adjacency matrix *A* [45,46].

Inspired by this result, we consider now the general case. Consider constraints given by a set of empirical averages of observables, as explained in the previous section. The above example certainly fits this setting: just consider binary observables associated to each pair of states (i,j) that evaluates to 1 when a transition from state *i* to state *j* has been observed in the data. In our general setting, we assume that the chosen observables have a finite maximum range *R*. From these observables the energy function H of finite range *R* is built as a linear combination of these observables. Using this energy function we build a matrix denoted by $L_{H}$, so that for every $y,w\in S^{R}$ its entries are given as follows:

(12) $$L_{H}\left(y,w\right)=\left\{\begin{array}{cc} e^{H(y_{1}w_{1,R-1})}\; & \mathrm{if}\;y_{1,R-1}=w_{0,R-2}\\ 0,\; & \mathrm{otherwise}. \end{array}\right.$$

where $y_{1}w_{R-1}$ is the concatenated block built from $y_{1}$ and $w_{1,R-1}$. For observables of range one, the matrix above is defined as $L_{H}\left(y,w\right)=e^{H(y)}$. Assuming H>-∞, the elements of the matrix $L_{H}$ are non-negative. Furthermore, in every non trivial case, the matrix is primitive and satisfies the Perron-Frobenius theorem [44]. Denote by ρ the unique largest eigenvalue of $L_{H}$. Just as above, we denote by u and v the left and right eigenvectors of $L_{H}$ associated to ρ. Notice that $u_{i}>0$ and $v_{i}>0$, for all i∈S. The *free energy* associated to a transfer matrix is the logarithm of the unique maximum eigenvalue.

The matrix $L_{H}$ can be turned into a Markov matrix of maximum entropy. For a primitive matrix *M* with spectral radius ρ, and positive right eigenvector v associated to ρ, the stochastic matrix built from *M* is computed as follows:

$$S\left(M\right)=\frac{1}{\rho }D^{-1}MD\;,$$

where *D* is the diagonal matrix with entries $D_{ii}=v_{i}$. The MEMC transition matrix *P* and unique stationary probability measure π are explicitly given by

(13) $$P=S\left(L_{H}\right);\;\pi_{i}:=\frac{u_{i}\;v_{i}}{\langle u,v\rangle },\;\forall i\in S.$$

Note that when H=0, the MEMC is characterized by the Markov transition matrix with components [47]:

$$P_{ij}=\frac{A_{ij}v_{j}}{\rho v_{i}},$$

where *A* is the adjacency matrix.

#### 4.3.3. Finite Range Gibbs Measures

For a fixed energy function H of range R≥2, there is a unique stationary Markov measure μ for which there exist a constant γ≥1 such that [46],

(14) $$\gamma^{-1}\leq \frac{\mu [x_{1,n}]}{\exp(\sum_{k=1}^{n-R+1}H\left(x_{k,k+R-1}\right)-\left(n+R-1\right)F\left[H\right])}\leq \gamma ,$$

that attains the supremum (11). The measure μ, as defined by (14), is known in the symbolic dynamics literature as *Gibbs measure in the sense of Bowen* [48]. All MEMCs belong to this class of measures. Moreover, the classical Gibbs measures in statistical mechanics are particular cases of (14), when γ=1, F[H]=logZ and H is an energy function of range one, leading to an i.i.d stochastic process characterized by the product measure μ. In this case the following holds:

$$\mu \left(x\right)=\frac{e^{H(x)}}{Z}\;\forall x\in S;\;Z=\sum_{x\in S}e^{H(x)}.$$

The free energy that is defined here has a deep relationship with the free energy in thermodynamics. Consider a thermodynamic system in equilibrium. The Helmholtz free energy derived from the partition function as follows:

$$F\left(\beta \right)=-\beta^{-1}\log Z$$

where β=1/(kT) and *k* is Boltzmann’s constant and *T* is the temperature.

This quantity is related to the cumulant generating function for the energy. In the context of the maximum entropy principle, the physical temperature and the Boltzmann’s constant play no role, so usually both are considered equal to 1. From the free energy, all of the thermodynamic properties of the system can be obtained via its derivatives, examples are the internal energy, specific heat, and entropy. It is to be noted that the definition used in this tutorial for the free energy (11) follows from the conventions used in the field of thermodynamic formalism [41,45,46] and changes its sign with the usual convention in the field of statistical mechanics.

### 4.4. Example

We present here the toy example that we will use to explore statistical properties of spike trains using the non-equilibrium statistical physics approach. We present the transfer matrix technique to compute the Markov transition matrix, its invariant measure and free energy from a potential H.

Consider a range-2 potential with two neurons (N=2). We use the notation introduced in Section 2.1:

$$H\left(x^{0,1}\right)=h_{1}x_{0}^{1}x_{1}^{2}+h_{2}x_{0}^{2}x_{1}^{1}.$$

The state space of this problem is given by:

$$(\begin{array}{c} 0\\ 0 \end{array}),\;(\begin{array}{c} 0\\ 1 \end{array}),\;(\begin{array}{c} 1\\ 0 \end{array}),\;(\begin{array}{c} 1\\ 1 \end{array})\;.$$

The transfer matrix (12) associated to H is, in this case, a 4×4 matrix

$$L_{{\mathrm{xx}}^{\prime }}=(\begin{array}{cccc} 1 & 1 & 1 & 1\\ 1 & 1 & e^{h_{2}} & e^{h_{2}}\\ 1 & e^{h_{1}} & 1 & e^{h_{1}}\\ 1 & e^{h_{1}} & e^{h_{2}} & e^{h_{1}+h_{2}} \end{array})\;.$$

This matrix satisfies the hypothesis of the Perron-Frobenius theorem. The maximum eigenvalue is

$$\rho =\frac{1}{2}(3+e^{\left(h_{1}+h_{2}\right)}+\sqrt{5+4e^{h_{1}}+4e^{h_{2}}+2e^{\left(h_{1}+h_{2}\right)}+e^{\left(2h_{1}+2h_{2}\right)}})\;,$$

and the free energy

(15) F[H]=log(ρ).

## 5. Statistical Properties of Markov Maximum Entropy Measures

The procedure of finding a maximum entropy model gives us a full statistical model of the system of interest. In this section we discuss the added value that having such a model can provide.

### 5.1. Cumulants from Free Energy

All the statistical properties of the observables and their correlations can be obtained by taking the successive derivatives of the free energy with respect to the Lagrange Multipliers. This property explains the important role played by the free energy in the framework of MEMC. In general,

$$\frac{\partial^{n}F\left[H\right]}{\partial h_{k}^{n}}=\kappa_{n}\;\forall k\in \left\{1,...,K\right\},$$

where $\kappa_{n}$ is the cumulant of order *n* (Equation (5)). In particular, taking the first derivative:

(16) $$\frac{\partial F[H]}{\partial h_{k}}=E_{p}\left\{f_{k}\right\}\;\forall k\in \left\{1,...,K\right\},$$

where $E_{p}\left\{f_{k}\right\}$ is the average with respect to the maximum entropy distribution *p*, which is equal to the average value of $f_{k}$ with respect to the empirical measure. With Equation (16) the parameters of the MEMC can be fitted to be consistent with fixed average values of observables.

Suppose that we compute from data the average values of the following observables $\langle x_{0}^{1}x_{1}^{2}\rangle \;=\;0.1$ and $\langle x_{0}^{2}x_{1}^{1}\rangle =0.3$. We solve (16) (two equations and two unknowns) and obtain $h_{1}=-1.98306$ and $h_{2}=1.48406$. With these parameters, the following Markov transition matrix and invariant measure are obtained from (13):

$$P_{xx^{\prime }}=(\begin{array}{cccc} 0.232971 & 0.469441 & 0.0987018 & 0.198886\\ 0.115617 & 0.232971 & 0.216056 & 0.435357\\ 0.549892 & 0.15252 & 0.232971 & 0.0646176\\ 0.272896 & 0.0756914 & 0.509966 & 0.141446 \end{array})\;\pi \left(x\right)=(\begin{array}{c} 0.29102\\ 0.248443\\ 0.248443\\ 0.212095 \end{array})\;.$$

### 5.2. Fluctuation-Dissipation Relations

For a first-order stationary Markov chain, since each $X_{n},n\geq 1$ depends on its predecessor, this induces a non-zero time-correlation between $X_{n}$ and $X_{n+r}$, even when the distance *r* is greater than 1. This correlation, and more generally, time correlations between observables can be directly derived from the free energy. This relationship is usually referred to as Fluctuation-dissipation, and is also related to the linear response function that is presented in Section 5.7.

Let *P* be an ergodic matrix and indexed by the states in some finite set *E*, and π be its unique stationary measure. In this general context, for two real-valued functions that depend on a fixed finite number of components, we define the *n*–step correlation as

$$\begin{array}{c} C_{f,g}\left(n\right)=E_{\pi }\left(f\left(X_{0}\right)g\left(X_{n}\right)\right)-E_{\pi }\left(f\left(X_{0}\right)\right)E_{\pi }\left(g\left(X_{0}\right)\right)\;. \end{array}$$

In the particular case of MEMC with potentials of range R>1 there is a positive time correlation between pairs of observables $f(x_{n})$ and $g(x_{n+r})$. Suppose the correlations decay fast enough so that (at least)

$$\sum_{n=0}^{\infty }\left|C_{f,g}\left(n\right)\right|<\infty \;.$$

Then the following sum (known as the Green-Kubo formula [49]) converges and is non-negative:

(17) $$\sigma_{f_{k},f_{j}}^{2}=C_{f_{k},f_{j}}\left(0\right)+\sum_{r=1}^{\infty }C_{f_{k},f_{j}}\left(r\right)+\sum_{r=1}^{\infty }C_{f_{j},f_{k}}\left(r\right).$$

Additionally, it can be shown that the energy function and the free energy depends smoothly upon maximum entropy parameters. Moreover, the correlations between observables can be obtained from the free energy through:

$$\sigma_{f_{k},f_{j}}^{2}=\frac{\partial^{2}F\left[H\right]}{\partial h_{k}\;\partial h_{j}}=\frac{\partial \mu (f_{j})}{\partial h_{k}}.$$

The relationship between a correlation and a derivative of the free energy is called the fluctuation-dissipation theorem [50]. For a MEMC characterized by μ(P,π), the fluctuation-dissipation relationships can be obtained explicitly:

(18) $$\begin{array}{cc} \frac{\partial^{2}F\left[H\right]}{\partial h_{k}\;\partial h_{j}}= & E_{\mu }\left[f_{k}f_{j}\right]-E_{\mu }\left[f_{k}\right]E_{\mu }\left[f_{j}\right]+\sum_{r=1}^{\infty }\sum_{x,x^{\prime }\in S}\left(f_{k}\left(x\right)f_{j}\left(x^{\prime }\right)\pi_{x}P_{xx^{\prime }}^{r}-E_{\mu }\left[f_{k}\right]E_{\mu }\left[f_{j}\right]\right)\\ \; & +\sum_{r=1}^{\infty }\sum_{x,x^{\prime }\in S}\left(f_{j}\left(x\right)f_{k}\left(x^{\prime }\right)\pi_{x}P_{xx^{\prime }}^{r}-E_{\mu }\left[f_{k}\right]E_{\mu }\left[f_{j}\right]\right). \end{array}$$

For MEMC built from *K* observables, the correlations can be conveniently arranged in a K×K symmetric matrix denoted by χ (the symmetry refers to the Onsager reciprocity relations [51]).

(19) $$\chi_{jk}=\frac{\partial^{2}F\left[H\right]}{\partial h_{k}\;\partial h_{j}}=\frac{\partial \mu (f_{j})}{\partial h_{k}}=\frac{\partial \mu (f_{k})}{\partial h_{j}}=\chi_{kj}.$$

For the example Section 4.4, we obtain the matrix χ by taking the second derivatives of (15) and evaluate at the parameters found previously,

$$\chi_{kj}=(\begin{array}{cc} 0.0971481 & 0.0606071\\ 0.0606071 & 0.127964 \end{array})\;.$$

**Figure 2:**
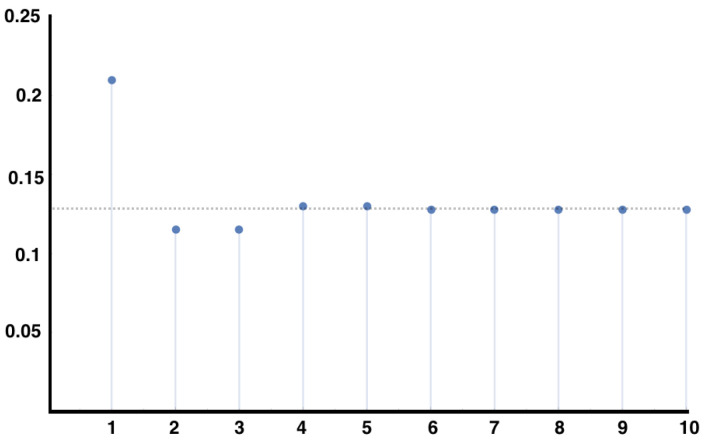
Plot of the auto-correlation of the observable $x_{0}^{2}x_{1}^{1}$ with respect to the MEMC consistent with constraints $\langle x_{0}^{1}x_{1}^{2}\rangle =0.1$ and $\langle x_{0}^{2}x_{1}^{1}\rangle =0.3$. The plot show the sum of Equation (18) from r=1 up to the number in the abscissa. Note the fast convergence towards $\chi_{22}$.

In Figure 2, we plot the right hand side of Equation (18) for the MEMC built from the example Section 4.4 consistent with constraints considered in the example of Section 5.1, for the auto-correlation of the observable $x_{0}^{2}x_{1}^{1}$.

### 5.3. Resonances and Decay of Correlations

We now turn back to the general setting of an arbitrary ergodic matrix *P* with stationary measure π associated to a Markov chain taking values on a finite state space (not necessarily the space of spike-patterns). Without loss of generality, assume that *P* is indexed by the states in E={1,2,…,M}. It can be proved that in this case there exists $\left(l_{i}:i=1,2,\ldots ,M\right)$ and $\left(r_{i}:i=1,2,\ldots ,M\right)$, sets of left and right eigenvectors respectively, associated to the eigenvalues $\left(\rho_{i}:i=1,\ldots ,M\right)$. We can assume that the eigenvectors and left and right eigenvalues have been sorted and normalized in such a way that $\rho_{1}=1$, $l_{1}$ is the unique *P*–stationary probability vector π, $r_{1}={\left(111\ldots 1\right)}^{T}$, and

$$\begin{array}{c} \langle l_{i}|r_{j}\rangle =\delta_{i,j}, \end{array}$$

where $\delta_{i,j}$ is the Kronecker delta, and 〈uv〉=〈u,v〉 corresponds to the Dirac’s bra-ket, $\left|u\rangle \langle v\right|=uv^{T}$. With the same notation, the spectral decomposition of *P* is written:

$$\begin{array}{c} P=\sum_{i=1}^{M}\rho_{i}|r_{i}[l_{i}]. \end{array}$$

Hence:

(20) $$\begin{array}{c} P^{n}=\sum_{i=1}^{M}\rho_{i}^{n}|r_{i}[l_{i}]. \end{array}$$

Given two functions f:E↦R and g:E↦R the following holds,

(21) $$\begin{array}{cc} C_{f,g}\left(n\right) & :=E_{\pi }\left(f\left(X_{0}\right)g\left(X_{n}\right)\right)-E_{\pi }\left(f\left(X_{0}\right)\right)E_{\pi }\left(g\left(X_{0}\right)\right)\\ \; & =\langle \pi f\circ P^{n}g\rangle -\langle \pi f\rangle \langle \pi g\rangle . \end{array}$$

Recall the discussion in previous sections regarding the reverse chain Section 2.4. Writing $E_{\pi }^{\leftarrow }$ for the expectation operator associated to the reverse Markov measure, i.e., to the measure $\mu =\mu (\pi ,\overset{\leftarrow }{P})$, one can see that

$$\begin{array}{c} E_{\pi }\left(f\left(X_{0}\right)g\left(X_{n}\right)\right)=E_{\pi }^{\leftarrow }\left(f\left(X_{n}\right)g\left(X_{0}\right)\right)\;, \end{array}$$

and hence (21) becomes

$$\begin{array}{c} \langle \pi |g\circ {\overset{\leftarrow }{P}}^{n}f\rangle -\langle \pi |f\rangle \langle \pi |g\rangle . \end{array}$$

From (20)

$$\begin{array}{c} f\circ P^{n}g=\sum_{i=1}^{M}\langle l_{i}|g\rangle |f\circ r_{i}\rangle \end{array}$$

and thus (21) becomes

(22) $$\begin{array}{cc} C_{f,g}\left(n\right) & =\sum_{i=1}^{M}\rho_{i}^{n}\langle l_{i}|g\rangle \langle \pi |f\circ r_{i}\rangle -\langle \pi |f\rangle \langle \pi |g\rangle \\ \; & =\sum_{i=2}^{M}\rho_{i}^{n}\langle l_{i}|g\rangle \langle \pi |f\circ r_{i}\rangle . \end{array}$$

**Figure 3:**
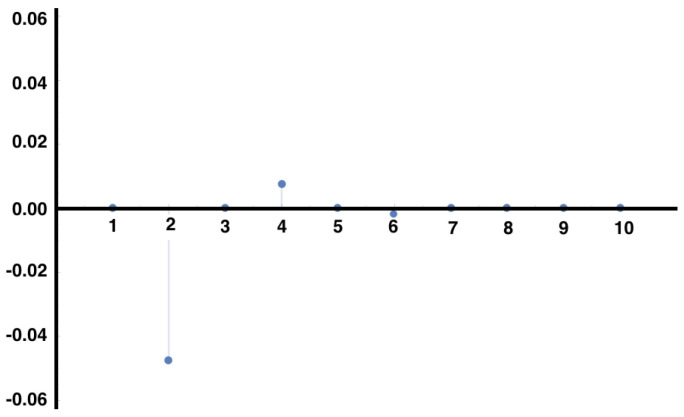
Auto-correlations of the observable $x_{0}^{2}x_{1}^{1}$ for the MEMC with the same parameters as in Figure 1. Modulations in the decay of correlations are due to the complex eigenvalues of the MEMC.

In Figure 3, we show the auto-correlations of the same observable considered in Figure 1, for the same MEMC. We observe modulations in the decay of the auto-correlations due to the complex eigenvalues in Equation (22), which arise in the non-symmetric transition matrix induced by the irreversibility of the MEMC.

We have found in Equation (22) an explicit expression for the decay of correlation for observables from the set of eigenvalues and eigenvectors of the transition matrix *P*. This is relevant in the context of spike train statistics because as the matrix *P* characterizing the spike trains is not expected to be symmetric, its eigenvalues are not necessarily real and modulations in the decay of correlations are expected (resonances). When measuring correlations between observables from data, one may observe this oscillatory situation that resembles resonances. This may be a symptom of a non-equilibrium situation.

### 5.4. Large Deviations for Average Values of Observables in MEMC

Obtaining the probability of “rare” average values of firing rates, pairwise correlations, triplets or non-synchronous observables is relevant in spike train statistics as these observables are likely to play an important role in neuronal information processing, and rare values may convey crucial information or be a symptom that the system in not working properly.

Here, we build from a previous article [52] where it is shown that the SCGF (7) can be obtained directly from the inferred Markov transition matrix *P* through the Gärtner-Ellis theorem (8). Consider a MEMC with transition matrix *P*. Let *f* be an observable of finite range and k∈R. We introduce the *tilted transition matrix by f* of *P*, parametrized by *k* and denoted by ${\tilde{P}}^{\left(f\right)}\left(k\right)$ [53] as follows:

(23) $${\tilde{P}}_{ij}^{\left(f\right)}\left(k\right)=P_{ij}e^{kf(ij)}\;i,j\in S.$$

The tilted transition matrix can be directly obtained from the spectral properties of the transfer matrix (12),

$$\begin{array}{ccc} {\tilde{P}}_{ij}^{\left(f\right)}\left(k\right) & = & \frac{e^{H_{ij}}v_{j}}{v_{i}\;\rho }e^{kf(ij)}\\ & = & \frac{e^{\left[H_{ij}+kf\left(ij\right)\right]}v_{j}}{v_{i}\;\rho }\;i,j\in S. \end{array}$$

Recall that v is the right eigenvector associated to its maximum eigenvalue ρ of the transfer matrix L. Here we use the notation $H_{ij}$ to specify that the energy function is built from the elements of the state space *i* and *j*. Remarkably, this result is valid not only for the observables in the energy function, i.e., from here the LDP of more general observables can be computed.

To obtain an explicit expressions for the SCGF $\lambda_{f}\left(k\right)$, it is possible to take advantage of the structure of the underlying stochastic process. For instance, for i.i.d. random process $X_{t}$ where $X_{i}∼X$ from Definition 7, one can obtain that

$$\lambda \left(k\right)=\lim_{t\to \infty }\frac{1}{t}\ln E{\left[e^{tkA_{t}\left(f\right)}\right]}^{t}=\ln E\left[e^{kf(X)}\right],$$

which is the case of range one observables. Using the Equation (23), we obtain that the maximum eigenvalue of the tilted matrix $\rho ({\tilde{P}}_{f}\left(k\right))$ is,

$$\rho ({\tilde{P}}_{f}\left(k\right))=\sum_{j}\pi_{j}e^{kf(j)}\;j\in S.$$

As ${\tilde{P}}_{f}$ is a primitive matrix, the uniqueness of $\rho ({\tilde{P}}_{f}\left(k\right))$ is ensured from the Perron-Frobenius theorem.

For additive observables of ergodic Markov chains, a direct calculation (see [54]) leads us to

$$\lambda_{f}\left(k\right)=\ln(\rho ({\tilde{P}}^{\left(f\right)})).$$

**Figure 4:**
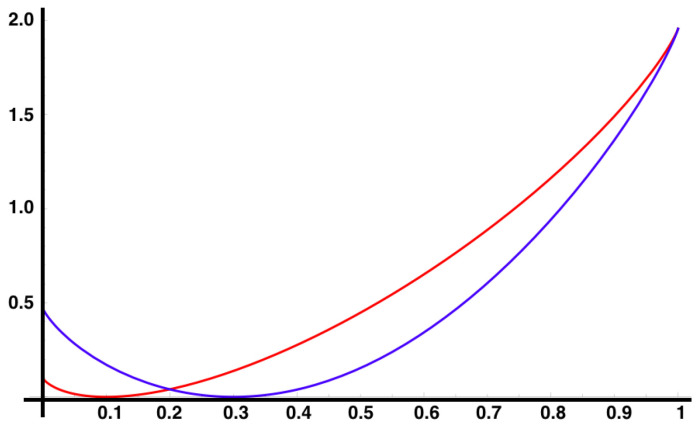
Rate functions of observables $x_{0}^{1}x_{1}^{2}$ in red, and $x_{0}^{2}x_{1}^{1}$ in blue for the MEMC consistent with constraints $\langle x_{0}^{1}x_{1}^{2}\rangle =0.1$ and $\langle x_{0}^{2}x_{1}^{1}\rangle =0.3$. The minimum value of both functions coincide with their expected values with respect to the MEMC. Around the minimum Gaussian fluctuations are expected (9). Far from the expected values are the large deviations.

It can also be proved that $\lambda_{f}\left(k\right)$, in this case, is differentiable [54], setting up the scene to use the Gärtner-Ellis theorem to obtain $I_{f}\left(s\right)$ as shown in Figure 4.

### 5.5. Information Entropy Production

Given a Markov chain $\left(X_{t}:t\geq 0\right)$ on a general finite state space *E* with transition matrix *P* started from the distribution ν, denoted $\nu^{\left(n\right)}$ the distribution of $X_{n}$, namely, for i∈E:

$$\begin{array}{c} \nu^{\left(n\right)}\left(i\right)=P_{\nu }\left(X_{n}=i\right)\;. \end{array}$$

Obviously, $\nu^{\left(0\right)}=\nu$, and

$$\begin{array}{c} \nu_{j}^{\left(n+1\right)}=\sum_{i\in E}\nu_{i}^{\left(n\right)}P_{ij}\;. \end{array}$$

The information-theoretic entropy of the probability distribution ν at time *n* is given by

$$\begin{array}{c} S_{n}\left(\nu \right):=-\sum_{i\in E}\nu_{i}^{\left(n\right)}\log\nu_{i}^{\left(n\right)}, \end{array}$$

and the *change of entropy* over one time step is defined as

$$\begin{array}{c} \Delta S_{n}:=S_{n+1}\left(\nu \right)-S_{n}\left(\nu \right). \end{array}$$

A bit of algebra yields

$$\Delta S_{n}=-\sum_{i,j\in E}\nu_{j}^{\left(n\right)}P_{ji}\log\frac{\nu_{j}^{\left(n+1\right)}P_{ji}}{\nu_{i}^{\left(n\right)}P_{ij}}+\frac{1}{2}\sum_{i,j\in E}\left[\nu_{j}^{\left(n\right)}P_{ji}-\nu_{i}^{\left(n\right)}P_{ij}\right]\log\frac{\nu_{j}^{\left(n\right)}P_{ji}}{\nu_{i}^{\left(n\right)}P_{ij}}.$$

The first term on the right hand side above is called *information entropy flow* and the second term *information entropy production* [12].

In the stationary case, i.e., when *P* admits a stationary measure π and the chain is started from that distribution, one has that $\nu^{\left(n\right)}=\pi$ for every n≥0; thus, in this case, the change of entropy rate is zero, i.e., for stationary chains, the information entropy flow equals (minus) information entropy production. This case is the focus of this work. The chain is associated to spike train activity for transitions between *L*–blocks. Starting from stationarity the entropy production rate is explicitly given by

(24) $$\begin{array}{c} IEP\left(P,\pi \right):=\frac{1}{2}\sum_{x,x^{\prime }\in S^{L}}\left[\pi \left(x^{\prime }\right)P_{x^{\prime }x}-\pi \left(x\right)P_{xx^{\prime }}\right]\log\frac{\pi \left(x^{\prime }\right)P_{x^{\prime }x}}{\pi \left(x\right)P_{xx^{\prime }}}\geq 0. \end{array}$$

The non-negativity implies that information entropy is positive as long as the process violates the detailed balance conditions (4). This is analogous to the second law of thermodynamics [55]. From this equation it is easy to realize that if the Markov chain satisfies the detailed balance condition, the information entropy production is zero.

**Figure 5:**
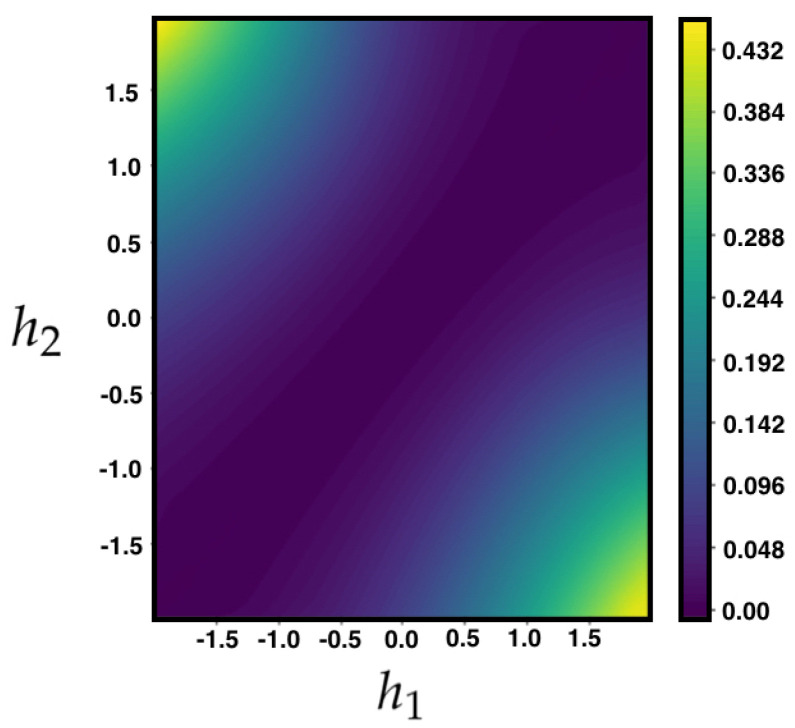
IEP for the MEMC of the example (Section 4.4) for different values of parameters $h_{1},h_{2}$. Observe that IEP(P,π)=0 when $h_{1}=h_{2}$ and that increases as the parameters become more different (more asymmetry in *P*).

In Figure 5, we compute the information entropy production from Equation (24), for the MEMC of the example (Section 4.4) for different values of the parameters $h_{1},h_{2}$.

It may seem contradictory that in stationary state the entropy is constant, while there is a positive “production” of entropy. The information entropy production in stationary state *always* compensate the information entropy flow, which leaves the information entropy rate constant. In this case we refer to non-equilibrium steady states (NESS).

### 5.6. Gallavotti-Cohen Fluctuation Theorem

To characterize the fluctuations of the IEP, consider the MEMC μ(P,π) and the following observable:

$$W_{n}\left(x_{0,n}\right)=\frac{1}{n}\ln\left(\frac{\mu (x_{0,n})}{\mu (x_{n,0})}\right),$$

It can be shown that $\lim\frac{1}{n}W_{n}\to IEP\left(\pi ,P\right)$. The Gallavotti-Cohen fluctuation theorem is as a statement about properties of the SCGF and rate function of the IEP [14].

(25) $$\lambda_{W}\left(k\right)=\lambda_{W}\left(-k-1\right),\;I_{W}\left(s\right)=I_{W}\left(-s\right)-s.$$

This symmetry holds for a general class of stochastic processes including NESS from Markov chains [56], and is a *universal property* of the IEP, i.e., it is independent of the parameters of the MEMC. To compute $\lambda_{W}\left(k\right)$ and $I_{W}\left(s\right)$, define $A{\left(k\right)}_{ij}=P_{ij}{\left[\frac{\pi_{i}P_{ij}}{\pi_{j}P_{ji}}\right]}^{k}$. If ρ(k) is the largest eigenvalue of A(k), then $\lim_{n\to \infty }\ln E\left(e^{n\lambda W_{n}}\right)=\ln\rho \left(k\right)$.

**Figure 6:**
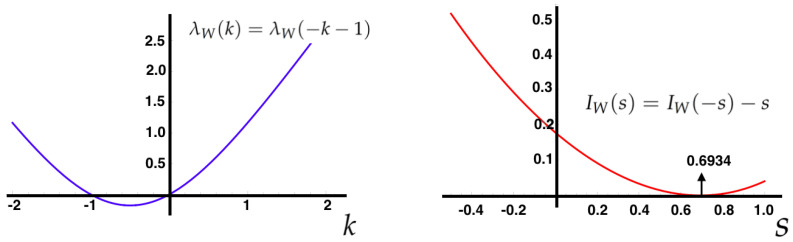
Gallavotti-Cohen symmetry property for the SCGF and rate function of the IEP (Equation (25)). Left: SCGF of the IEP of the MEMC with the same parameters considered in the previous examples. Right: Rate function of the observable *W*, the minimum is attained at the expected value of IEP.

In Figure 6, we illustrate the Gallavotti-Cohen symmetry property of the large deviation functions associated to the IEP (Equation (25)).

These properties are relevant to the large deviations of the averaged entropy production denoted $\frac{W_{t}}{t}$ over a trajectory $x_{0,t-1}$ of the Markov chain p(π,P). The following relationship holds,

$$\frac{p\left\{\frac{W_{t}}{t}\approx s\right\}}{p\left\{\frac{W_{t}}{t}\approx -s\right\}}≍e^{ts}.$$

This means that the positive fluctuations of $\frac{W_{t}}{t}$ are exponentially more probable than negative fluctuations of equal magnitude.

### 5.7. Linear Response

The linear response serves to quantify how a small perturbation δh of a set of the maximum entropy parameters affects the average values of observables in terms of the unperturbed measure. This is relevant in the context of spike trains statistics to identify stiff and sloppy directions in the space of parameters. A small change in a sloppy parameter produces very little impact in the statistical model. In contrast, a small change in a stiff parameter produces a significant change. For a MEMC characterized by μ=(P,π) corresponding to an energy function with fixed parameters h denoted by $H_{h}$, one can obtain the average value of a given observable $f_{k}$ from (16).

Now, consider a perturbed energy denoted by $\tilde{H}=H_{h+\delta h}$. Using a Taylor expansion, the average value of an arbitrary observable $f_{k}$ with respect to the MEMC can be obtained $\tilde{\mu }=\left(\tilde{P},\tilde{\pi }\right)$ associated to the perturbed energy. Considering the Taylor expansion of $F[H_{h+\delta h}]$ about $H_{h}$

(26) $$\begin{array}{ccc} \frac{\partial F[H_{h+\delta h}]}{\partial h_{k}} & = & \frac{\partial F[H_{h}]}{h_{k}}+\sum_{j}\frac{\partial^{2}F\left[H_{h}\right]}{\partial h_{k}h_{j}}\delta h_{j}+O{\left(\delta h_{j}\right)}^{2}\;, \end{array}$$

(27) $$\begin{array}{ccc} E_{\tilde{\mu }}\left[f_{k}\right] & = & E_{\mu }\left[f_{k}\right]+\sum_{j}\frac{\partial^{2}F\left[H_{h}\right]}{\partial h_{k}h_{j}}\delta h_{j}+O{\left(\delta h_{j}\right)}^{2}\;, \end{array}$$

(28) $$\begin{array}{ccc} \Delta E[f] & \approx & \chi \cdot \delta h\;. \end{array}$$

**Figure 7:**
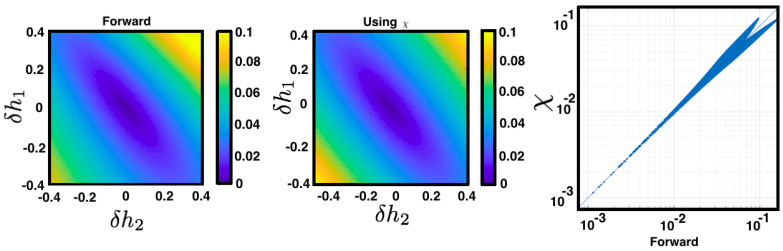
Linear response for the MEMC of the example (Section 4.4) for different values of perturbations $\delta h_{1}$ and $\delta h_{2}$. The colors represent $∥E_{\tilde{\mu }}\left[f_{k}\right]-E_{\mu }\left[f_{k}\right]∥$ computed using two methods. The “forward” method consists in computing $E_{\tilde{\mu }}\left[f_{k}\right]$ from $\tilde{\mu }$ and $E_{\mu }\left[f_{k}\right]$ from μ. The figure in the middle is obtained by computing $∥E_{\tilde{\mu }}\left[f_{k}\right]-E_{\mu }\left[f_{k}\right]∥$ from χ using Equation (28). (**Right**) The difference between both methods illustrated in a scatter plot in logarithmic scale.

We use (16) to go from (26) to (27). Observe from (27) that a small perturbation of a parameter $h_{j}$ influences the average value of all other observables in the energy function (as $f_{k}$ is arbitrary). The perturbation is modulated by the second derivatives of the free energy corresponding to the unperturbed regime $F[H_{h}]$ (see Figure 7).

## 6. Discussion and Future Work

This tutorial explores how one can use maximum entropy methods to capture asymmetric temporal aspects of spike trains from experimental data. In particular, we showed how spatio-temporal constraints can produce homogeneous irreducible Markov chains whose unique steady state is, in general, non-equilibrium (NESS)—thus, detailed balance condition is not satisfied causing strictly positive entropy production. This fact highlights that only non-synchronous maximum entropy models induce time irreversible processes, which is one of the key hallmarks of biological systems.

We have presented a survey of diverse techniques from mathematics and statistical mechanics to study these NESS, which correspond to a rich toolkit that can be employed to study unexplored aspects of spike train statistics. We emphasise that many of these concepts, including entropy production and fluctuation-dissipation relationships, have not been explored much in the context of spike train analysis. However, the fact that time irreversibility is such an important feature of living systems suggest that these notions might play an important role in neural dynamics.

Possible extensions include measuring the entropy production for different choices of spatio-temporal constraints using the maximum entropy method on biological spike train recordings. A more ambitious extension is to explore the relationship between entropy production computed from experimental data obtained from different physiological processes and relate them to features such as adaptation or learning. Concerning time-dependent neuronal network models, future studies might lead to a better understanding of the impact of particular synaptic topologies of neuronal network models on the corresponding entropy production, decay of correlations, resonances and other sophisticated statistical properties.

Other possible extensions are related to the drawbacks of current approaches. This can include limitations of the maximum entropy method related to the requirement of stationarity in the data, which is not a natural condition for some biological scenarios. However, several of the techniques presented in this tutorial naturally extend to the non-stationary case, including the information entropy production, which can still be defined along non-stationary trajectories [14]. Related to this issue is that the approach presented in this tutorial does not make any reference to the stimulus. While this issue has been addressed in the synchronous framework [34], there is still an open field to explore the Markovian extension of these ideas. Another interesting topic to explore in future studies is the inclusion of the non-stationary approach such as the state space analysis proposed in [35]. Also, another open problem is related to the efficient implementation of the transfer matrix technique, which currently requires an important computational effort in the case of large neural networks. Recently, some improvements of this approach have been proposed based in Monte Carlo methods [57].

In summary, we believe that these topics are fertile ground for multi-disciplinary exploration by teams composed of mathematicians, physicists, and neuroscientists. It is our hope that this work may foster future collaborative research among disciplines, which might bring new breakthroughs to advance our fundamental understanding of how the brain works.

## Abbreviations

The following abbreviations are used in this manuscript:

MEP	Maximum entropy principle
MEMC	Maximum entropy Markov chain
SCGF	Scaled cumulant generating function
CLT	Central limit theorem
LLN	Law of large numbers
LDP	Large deviation principle
IEP	Information entropy production
KSE	Kolmogorov-Sinai entropy
NESS	Non-equilibrium steady states
**Symbol list**	
S	${\left\{0,1\right\}}^{N}$ the state space of spike patterns of *N* neuron
Ω	The set of infinite sequences of spike patterns
$x_{n}^{k}$	Spiking state of neuron *k* at time *n*
$x_{n}$	Spike pattern at time *n*
$x_{t_{1},t_{2}}$	Spike block from time $t_{1}$ to $t_{2}$
ν(f)	Expectation of the observable *f* w.r.t. the probability measure ν
$A_{T}\left(f\right)$	Empirical Average value of the observable *f* considering *T* spike patterns
$S^{R}$	Space of spike blocks of *N* neurons and length *R*
S[μ]	Entropy of the probability measure μ
H	Energy function
F[H]	Free energy

## References

[1] Rieke F., Warland D., de Ruyter van Steveninck R., Bialek W. (1996). Spikes, Exploring the Neural Code.

[2] Bialek W. (2012). Biophysics: Searching for Principles.

[3] Schneidman E., Berry M.J., Segev R., Bialek W. (2006). Weak pairwise correlations imply strongly correlated network states in a neural population. Nature.

[4] Ganmor E., Segev R., Schneidman E. (2011). Sparse low-order interaction network underlies a highly correlated and learnable neural population code. Proc Natl. Acad. Sci. USA.

[5] Tkačik G., Marre O., Amodei D., Schneidman E., Bialek W., Berry M.J. (2014). Searching for collective behavior in a large network of sensory neurons. PLoS Comput. Biol..

[6] Palsso B. (2006). Systems Biology: Properties of Reconstructed Networks.

[7] Tang A., Jackson D., Hobbs J., Chen W., Smith J., Patel H., Prieto A., Petrusca D., Grivich M., Sher A. (2008). A maximum entropy model applied to spatial and temporal correlations from cortical networks in vitro. J. Neurosci..

[8] Marre O., El Boustani S., Frégnac Y., Destexhe A. (2009). Prediction of spatiotemporal patterns of neural activity from pairwise correlations. Phys. Rev. Lett..

[9] Vasquez J., Palacios A., Marre O., Berry M., Cessac B. (2012). Gibbs distribution analysis of temporal correlation structure on multicell spike trains from retina ganglion cells. J. Physiol. Paris.

[10] Mora T., Deny S., Marre O. (2015). Dynamical criticality in the collective activity of a population of retinal neurons. Phys. Rev. Lett..

[11] Cofré R., Cessac B. (2014). Exact computation of the maximum entropy potential of spiking neural networks models. Phys. Rev. E.

[12] Cofré R., Maldonado C. (2018). Information entropy production of maximum entropy Markov chains from spike trains. Entropy.

[13] Schulman L.S. (1997). Time’s Arrows and Quantum Measurement.

[14] Jiang D.Q., Qian M., Qian M.P. (2004). Mathematical Theory of Non-Equilibrium Steady States.

[15] Schrödinger E. (1944). What Is Life? The Physical Aspect of the Living Cell.

[16] Prigogine I. (1962). Nonequilibrium Statistical Mechanics.

[17] Deem M. (2007). Mathematical adventures in biology. Phys. Today.

[18] Filyukov A., Karpov V. (1967). Description of steady transport processes by the method of the most probable path of evolution. Inzhenerno-Fizicheskii Zhurnal.

[19] Filyukov A., Karpov V. (1967). Method of the most probable path of evolution in the theory of stationary irreversible processes. Inzhenerno-Fizicheskii Zhurnal.

[20] Favretti M. (2009). The maximum entropy rate description of a thermodynamic system in a stationary non-equilibrium state. Entropy.

[21] Monthus C. (2011). Non-equilibrium steady states: maximization of the Shannon entropy associated with the distribution of dynamical trajectories in the presence of constraints. J. Stat. Mech. Theor. Exp..

[22] Shi P., Qian H., Feng J., Fu W., Sun F. (2010). Frontiers in Computational and Systems Biology.

[23] Galves A., Löcherbach E. (2013). Infinite systems of interacting chains with memory of variable length-A stochastic model for biological neural nets. J. Stat. Phys..

[24] Cofré R., Cessac B. (2013). Dynamics and spike trains statistics in conductance-based Integrate-and-Fire neural networks with chemical and electric synapses. Chaos Solitons Fractals.

[25] Halmos P.R. (1974). Measure Theory.

[26] Levin D., Peres Y. (2017). Markov Chains and Mixing Times.

[27] Gerstner W., Kistler W. (2002). Spiking Neuron Models.

[28] Jones G.L. (2004). On the Markov chain central limit theorem. Probab. Surv..

[29] Ellis R. (1985). Entropy, Large Deviations and Statistical Mechanics.

[30] Touchette H. (2009). The large deviation approach to statistical mechanics. Phys. Rep..

[31] Dembo A., Zeitouni O. (2010). Large deviations techniques and applications. Stochastic Modelling and Applied Probability.

[32] Marre O., Amodei D., Deshmukh N., Sadeghi K., Soo F., Holy T., Berry M. (2012). Mapping a complete neural population in the Retina. J. Neurosci..

[33] Tkačik G., Mora T., Marre O., Amodei D., Berry M., Bialek W. (2015). Thermodynamics for a network of neurons: Signatures of criticality. Proc Natl. Acad. Sci. USA.

[34] Granot-Atedgi E., Tkačik G., Segev R., Schneidman E. (2013). Stimulus-dependent maximum entropy models of neural population codes. PLoS Comput. Biol..

[35] Shimazaki H., Amari S., Brown E.N., Grün S. (2012). State-space analysis of time-varying higher-order spike correlation for multiple neural spike train data. PLoS Comput. Biol..

[36] Dewar R. (2003). Information theory explanation of the fluctuation theorem, maximum entropy production and self-organized criticality in non-equilibrium stationary states. J. Phys. A Math. Gen..

[37] Dewar R. (2005). Maximum entropy production and the fluctuation theorem. J. Phys. A Math. Gen..

[38] Martyushev L., Seleznev V. (2006). Maximum entropy production principle in physics, chemistry and biology. Phys. Rep..

[39] Jaynes E. (1980). The minimum entropy production principle. Ann. Rev. Phys. Chem..

[40] Pressé S., Ghosh K., Lee J., Dill K.A. (2013). Principles of maximum entropy and maximum caliber in statistical physics. Rev. Mod. Phys..

[41] Ruelle D. (1978). Thermodynamic Formalism.

[42] Jaynes E. (1957). Information theory and statistical mechanics. Phys. Rev..

[43] Tkačik G., Marre O., Mora T., Amodei D., Berry M., Bialek W. (2013). The simplest maximum entropy model for collective behavior in a neural network. J. Stat. Mech..

[44] Seneta E. (2006). Non-Negative Matrices and Markov Chains.

[45] Walters P. (1975). Ruelle’s operator theorem and *g*-measures. Trans. Am. Math. Soc..

[46] Bowen R. (2008). Equilibrium States and the Ergodic Theory of Anosov Diffeomorphisms. Lecture Notes in Mathematics.

[47] Parry W., Pollicott M. (1990). Zeta functions and the periodic orbit structure of hyperbolic dynamics. Astérisque, Société mathématique de France.

[48] Chazottes J., González-Aguilar H., Ugalde E. (2015). Fluctuations of observables in dynamical systems: From limit theorems to concentration inequalities. Nonlinear Dynamics New Directions.

[49] Gaspard P. (1998). Chaos, Scattering and Statistical Mechanics.

[50] Bettolo U.M., Puglisi A., Rondoni L., Vulpiani A. (2008). Fluctuation–dissipation: Response theory in statistical physics. Phys. Rep..

[51] Gaspard P. (2014). Random paths and current fluctuations in nonequilibrium statistical mechanics. J. Math. Phys..

[52] Cofré R., Maldonado C., Rosas F. (2018). Large deviations properties of maximum entropy Markov shains from spike trains. Entropy.

[53] Touchette H. (2012). A Basic Introduction to Large Deviations: Theory, Applications, Simulations. arXiv.

[54] Ellis R.S. (2010). The theory of large deviations and applications to statistical mechanics. Long-Range Interacting Systems.

[55] Nicolis G., Nicolis C. (2012). Foundations of Complex Systems: Emergence, Information and Prediction.

[56] Maes C. (1999). The fluctuation theorem as a Gibbs property. J. Stat. Phys..

[57] Nasser H., Cessac B. (2014). Parameter estimation for spatio-temporal maximum entropy distributions: Application to neural spike trains. Entropy.

